# First insights into multidisciplinary and multispecialty long COVID networks—a SWOT analysis from the perspective of ambulatory health care professionals

**DOI:** 10.3389/fmed.2023.1251915

**Published:** 2023-11-09

**Authors:** Sandra Stengel, Lea Gölz, Joachim Kolb, Karin Tarbet, Stefanie Völler, Jan Koetsenruijter, Joachim Szecsenyi, Uta Merle

**Affiliations:** ^1^Department of General Practice and Health Services Research, Faculty of Medicine, University Hospital Heidelberg, Heidelberg, Germany; ^2^General Practice, Benningen am Neckar, Germany; ^3^Department of Internal Medicine IV, University Hospital Heidelberg, Heidelberg, Germany; ^4^Institute of General Practice and Interprofessional Care, University Hospital Tübingen, Tübingen, Germany

**Keywords:** long COVID, network, ambulatory care, multispecialty, multidisciplinary, SWOT analysis, resilience

## Abstract

**Introduction:**

Multidisciplinary and multispecialty approaches with central integration of primary care, individualized long-term rehabilitative care, and multidisciplinary care pathways are recommended by international consortia to face the challenges of care of long COVID. Two regional long COVID networks—Rhein-Neckar (RN) and Ludwigsburg (LU) have emerged as *ad hoc* examples of best practice in Southern Germany. The aim of the community case study is to provide first insights into the experiences of the networks.

**Methods:**

The exploratory observational study was conducted between April and June 2023, focusing on an observation period of just under 24 months and using a document analysis supported by MAXQDA and SWOT analysis with ambulatory health care professionals in two online group discussions.

**Results:**

The document analysis revealed that both networks have defined network participants who have agreed on common goals and patient pathways and have established ways of communicating, organizing, and collaborating. Both networks agreed on a primary care-based, multidisciplinary and multispecialty approach. The main differences in realization emerged in LU as a focus on the ambulatory setting and very concrete application to individual patients, while RN showed a focus on an intersectoral character with participation of the specialized university hospital sector, knowledge transfer and a supra-regional approach with the involvement of the meso and macro level. The SWOT analysis (*n* = 14 participants, *n* = 6 male, 7 physicians (4 disciplines), 7 therapists (5 professions)) showed strengths such as resulting collaboration, contribution to knowledge transfer, and improvement of care for individual patients. As barriers, e.g., lack of reimbursement, high efforts of care, and persistent motivation gaps became apparent. Potentials mentioned were, e.g., transferability to other diseases such as Myalgic Encephalomyelitis/Chronic Fatigue Syndrome, promotion of addressing a “difficult topic” and promotion of intersectoral care concepts; risks mentioned were, e.g., limited network resources and negative effects on the development of other structures.

**Conclusion:**

Resulting implications for practice and research address a call to policy makers and funders to support further research to find out what generalizable results regarding usefulness, effectiveness, and efficiency including transferability to other post-infectious diseases can be derived.

## 1. Introduction

More than 3 years after the start of the SARS-CoV-2 pandemic, the World Health Organization (WHO) declares the end of the COVID-19 emergency phase (1), while at the same time the consequences pose continuing major challenges to health systems: pandemic-related, with 767 million confirmed SARS-CoV-2 infections worldwide (2) a large number of people are simultaneously affected by post-acute sequelae of SARS-CoV-2 infection, with an estimated 17 million people affected in Europe alone (3). Data on prevalence are still inconclusive and vary due to heterogeneous study designs and different subgroups (4) but still show persistent symptoms in a relevant number of cases at 1 year (5). Persistent symptoms following SARS-CoV-2 infection with no other identifiable cause are referred to as Acute COVID up to 4 weeks, Long COVID beyond 4 weeks, and Post COVID beyond 3 months (6, 7). In health care settings, because patients may contact the health care system at any time due to SARS-CoV-2 infection, we use the broader term Long COVID below for persistent symptoms.

The lack of knowledge and acceptance among healthcare providers and as the resulting underuse of care are well documented in Long COVID internationally and in Germany (8, 9). Challenges of the Long COVID care are on the one hand the rapid generation of knowledge with currently 15.648 hits in a PubMed search on 7 June 2023 (“post covid” OR “long covid” OR “PACS”) and on the other hand the lack of clinically relevant evidence on pathogenesis, diagnosis and therapy (10–13), resulting in holistic, currently symptom-oriented therapeutic approaches outside of trials (7, 14, 15). The translation of knowledge from research to practice as a key implementation component for improved care is therefore all the more urgent in this dynamic field. Increased use in primary care has been described internationally (16). Less evidence is found from the perspective of health care providers, for example, among other things, a lack of competence in long COVID, resulting uncertainty (9), a high time commitment, and a desire for supportive primary care interventions (17).

Positive and negative experiences with the health care system reported by patients can be used to develop care models. For example, the desire for face-to-face services and multidisciplinary, holistic services from a single source (“one-stop clinics”) was addressed in a qualitative systematic review (8). Patients and general practitioners from the Rhine-Neckar region in Germany also expressed the need for a structured overall concept with competent contact points and coordination of medical care in Long-COVID (9). Multidisciplinary and multispecialty approaches with central integration of primary care, individualized long-term rehabilitative care, and multidisciplinary care pathways are recommended by international consortia (18, 19) and being established as best practice worldwide (20–23). In Germany, both health professionals and patient representatives have called for the establishment of networks for this purpose (24, 25). In Germany, the ambulatory sector is well developed with a comprehensive range of practices with physicians (general practitioners and specialists), occupational therapists, speech therapists, physiotherapists and psychotherapists (26). A main challenge is the separated organization and governance of the health care sectors and resulting fragmentation of health care (27). Building on the experience of intersectoral networking during the acute COVID pandemic (28) and incorporating the results of the aforementioned regional survey of support and care needs (9), a regional “competence network Long COVID Rhein-Neckar” (RN) was established as part of a funded project under the direction of the University Hospital Heidelberg, in collaboration with the “Departments of General Practice and Health Services Research” and the “Internal Medicine—Department for Gastroenterology, Infectious Diseases, Toxicology” with the offer of a post COVID outpatient clinic (29). RN was in exchange with the “Long COVID network Ludwigsburg” as an informal association without funding (LU), which was established by the medical profession in Ludwigsburg, a district about 100 km away (30). The term network is used in reference to Gamper: “Networks are made up of actors who are connected to each other through relationships, and whose connections come together to form different social structures” (31). The networks have emerged as *ad hoc* examples of best practice, formed in a pragmatic way in response to the pressure of the situation.

The aim of the community case study is to provide first insights into the experiences of the two regional Long COVID networks in Southern Germany, which have been set up as *ad hoc* examples of best practice. The following questions should be answered:

- How are the networks structured and how do they work?
- What are the strengths, weaknesses, opportunities and risks of the networks from the perspective of the participating ambulatory health care professionals?

## 2. Methods

### 2.1. Study design and setting

The exploratory observational study was conducted between April and June 2023 after receiving a positive ethics approval from the Ethics Committee of Heidelberg University Hospital (S-233/2023, April 26, 2023), using a document analysis (32) and SWOT analysis in an online group discussion as a business method for identifying a company’s strategic need for action (33). Its use is also well established in medicine (34–36). The observation period within the document analysis and SWOT analysis performed covers the start dates of the networks (RN May 2021; LU January 2022) until the implementation of the study, the start of which was defined by the presence of a positive ethics vote in April 2023.

### 2.2. Recruitment and sample

For the document analysis, the documents created during the establishment and realization of the network activities were retrospectively evaluated. The documents were publicly available on the respective websites. Other documents, such as minutes and internal progress reports, were analyzed retrospectively in an anonymized form after receiving the ethics vote.

The target group of the SWOT analysis were the network partners of RN and LU, who offer a health and/or care service in the ambulatory sector, or patients who are involved in the network advisory board/in the network organization. The target group of both networks (*n* = 23 Rhein-Neckar; *n* = 52 Ludwigsburg) was contacted by e-mail and invited to participate in the SWOT analysis. A reminder was sent after a few days. In case of interest, an information leaflet and a consent form were sent. Information about the study procedure was provided verbally and open questions could be asked and clarified. In case of consent, socio-demographic data (gender, role in the network, rural/urban work location) were pseudonymized. Consent to participate was given in writing. In case of more than eight interested persons per network, a purposive sampling strategy was planned. As this number was not reached in either network, all interested participants were invited to participate. An incentive of € 150 was paid for participation in the SWOT analysis in an online group discussion (90 min).

### 2.3. Data collection and analysis

In the case of RN, in the document analysis an internal progress report, programs of the training courses, protocols of the advisory board meetings and working groups, and contents of the website were evaluated (29). In LU, relevant documentation from the Rhein-Neckar internal progress report, the protocol of the introduction of the SWOT group discussion, the internal documentation from LU, and website content (30) were included in the analysis. In the document analysis, using a combination of qualitative content analysis and thematic analysis with support of MAXQDA, topics for the presentation of the networks were identified by SS in an iterative process and discussed with LG (master’s student of health services research and implementation science, experience in qualitative research). The contents were integrated by SS (general practitioner, experienced qualitative researcher of the study team and coordinator of RN) supported by the main coordinator of LU (JK) based on the results of the document analysis in an iterative process. The identified topics and contents were additionally checked for plausibility in the network coordination (UM, gastroenterologist, experienced researcher of the study team and coordinator of RN) and for comprehensibility in the study team (LG, SV).

The SWOT analysis was moderated in an online group discussion by a member of the study team and recorded pseudonymously by another person. Due to the coordinating role of SS in RN, she did not participate in the corresponding regional group discussion in order to avoid social desirability. The group discussion was structured as follows: 1. exchange in small groups as introduction; 2. input to the SWOT analysis; 3. SWOT analysis in small groups with documentation in a 4-field board; 4. presentation of the SWOT analysis results of the groups; 5. common discussion of the synopsis of the analysis results as well as completion of the documentation if necessary. From the documentation of the group discussions (SWOT documentation and protocol of the discussion), topics were 1. merged and 2. clustered by SS. Subsequently, the result was checked for comprehensibility by LG and aspects with difficulties in understanding and/or comprehensibility were discussed point by point and agreed between SS and LG. Finally, the synthesized document was sent to the group of participants in the form of a member check. The original SWOT documentation was added. As reflection questions were asked: “1. In your opinion, has something important been lost? If so, please name the aspect(s). 2. Is there anything mentioned in the synthesized version that you see as a wrong result of the group discussion? If so, please identify this aspect/these aspects. Please only comment on what was documented in the group discussion and do not add any new aspects.” The feedback was discussed, agreed upon and integrated between LG and SS.

## 3. Results

### 3.1. Network establishment and realization

Identified categories from the document analysis were start, sponsoring, build-up, coordination, consented goals, definition, activity status, activities, and treated patients.

Table 1 shows the characteristics of the two networks RN and LU. RN started on May 22, 2021, LU started on January 01, 2022. There were similarities and differences, but the latter predominated: Similarities can be seen in the definition of the network. Both networks defined network participants who have agreed on common goals as well as patient pathways and have established ways of communicating, organizing and collaborating. On the one hand, identified network partners in both networks are listed on a website and are thus contact persons for medical care in Long COVID; on the other hand, general practitioners in both networks are responsible for basic care and care coordination. In RN, patient representation was continuously involved in the coordination of the network through the advisory board via self-help group members; in LU, the network was defined by stakeholders. In addition, perceived care needs were the trigger for the establishment of the networks in both regions. There were also similarities in the agreed objectives, such as avoiding underuse and overuse. The regional collaboration included working groups, advisory board meetings, participation of regional stakeholders in training programs (RN) and quality circles, interface agreements within the care process, and participation of regional actors in training programs (LU). At the time of manuscript preparation in May 2023, there were approximately the same number of listed network partners with multidisciplinary and multispecialty composition, i.e., 34 (RN) or 36 (LU) stakeholders who are actively involved in the care of patients with long COVID. Differences can be seen in the start of network activity, which started 7 months earlier in RN than in LU. RN received project funding that included network coordination and reimbursement for SWOT analysis, whereas LU was carried out solely on a voluntary basis. In both networks, care was provided within the standard of care without additional incentive. The perceived need that triggered the establishment of the network in RN was from an inpatient perspective (that means special ambulatory department for Long COVID of the university hospital) and in LU from an ambulatory perspective, which continued in the further establishment and coordination. In the consensual objectives, RN showed a more provider-oriented perspective, whereas in LU the patient level was also taken into account. In the category definition of the network, the unique selling point in RN was the formulated conditions of participation and the presentation of an interdisciplinary, interprofessional and intersectoral advisory board with integration of the meso level; in LU, more concrete and specific coordinated agreements for the interfaces emerged. The activity status in May 2023 shows differences between the regions. The project character in RN includes on the one hand an end of the project and on the other hand the prospect of a continuation in a follow-up project. In LU there is a continuing activity without funding. Differences are still evident in the intersectoral focus in RN and the ambulatory focus in LU.

**Table 1:** Network characteristics.

Competence network Long COVID Rhein-Neckar	Long COVID network Ludwigsburg
**Start**	
- May 22, 2021	- January 01, 2022
Funding	
- Realization MWK^1^: Personnel costs in the university setting and compensation for participation in self-help. - Evaluation MWK^1^: Reimbursement of expenses for participation in the SWOT analysis	- Realization: none - Evaluation MWK^1^: Reimbursement of expenses for participation in the SWOT analysis
**Structure**	
- Trigger: Appointment requests at the University’s Long COVID Outpatient Clinic that cannot be accommodated due to capacity constraints. - Continuation of an intersectoral cooperation between the Department of General Practice & Health Services Research and the Department of Internal Medicine IV/Long COVID Outpatient Clinic of Heidelberg University Hospital, which was established during the COVID-19 pandemic.^2^ - Identification of stakeholders and interested parties in a snowball system	- Trigger: Perceived needs in the ambulatory sector and response to closure of the Long COVID Outpatient Clinic at the nearby hospital. - Informal association of general practitioners, ambulatory specialists, and therapists as well as two rehabilitative institutions - Integrated into the regional “Quality in Ambulatory Medicine Working Group” (initiative of the ambulatory medical profession)
**Coordination**	
- General Practitioner from the Department of General Practice & Health Services Research (SS) and Specialist in Gastroenterology from the Department of Internal Medicine IV/Long COVID Outpatient Clinic of Heidelberg University Hospital (UM)	- General practitioner (JK)
**Consensus goals**	
- Provide long-term medical care services with sufficient capacity and based on current knowledge. - Share knowledge - Identify and communicate contacts - Coordinate patient pathways - Communicate information →Improve skills →Making the most of existing outpatient resources →Increase acceptance of the disease →Reduce uncertainties in treatment →Avoid both underuse and overuse	- Identification of patients in need of advanced or specialized diagnostic/therapeutic services - Provide specialized medical diagnostic and treatment services in an interdisciplinary network - Application of treatment methods according to indication and need, avoiding underuse, overuse or misuse - Fostering personal resources and resilience factors of patients to increase the ability to cope with everyday life and professional resilience. - Counteracting uncertainty, dysfunctional coping and chronification of symptoms - Network-wide incorporation of new knowledge and experience in diagnostics and therapy, and adaptation of network structures as required.
**Network definition**	
- Participation = Listing as network partner on the web site and/or participation in the advisory board. - Requirements for listing: 1. involvement in Long COVID medical care; 2. active participation in knowledge transfer or on the advisory board; and/or 3. certificate of participation in Long COVID continuing education. - Statement: Basic medical care is provided by any general practitioner, therefore no listing of general practitioners on the web site. - Orientation of care toward the consented care concept (general practitioners based medical care and coordination; stepped concept). - Advisory board: Multidisciplinary (medicine: general practice, gastroenterology, pediatrics, psychiatry, psychosomatics, rheumatology, sports medicine), multispecialty (occupational therapy, physiotherapy, psychotherapy), intersectoral (ambulatory and university hospital) providers; medical profession/medical association, association of statutory health insurance physicians, local authorities, health insurance funds, self-help groups of those affected.	- Participation = Listing as network partner on the web site - The network is defined by its aims - The coordination of diagnosis and treatment is carried out by the respective general practitioner or Long COVID specialized general practices based on of written treatment pathways and interface agreements between different medical groups and service providers. Each referral for co-treatment by a specialist must contain the complete results of the basic diagnostics, the current therapy and anamnestic information (“interface agreements”). The referral for medical or therapeutic co-treatment is made by the coordinating practice, stating the problem and the urgency (time frame).
**Activity status May 2023**	
- Project end date December 31, 2022 - Follow-up project with focus on supra-regional network ongoing with work package participatory regional network development in pilot regions - Web site is maintained by the Department of General Practice and Health Services Research Heidelberg, 34 network partners listed (26 ambulatory, 7 inpatient/university hospital), 11 specialties/professions	- 36 participating practices/facilities (ambulatory), 11 specialties/professions, 52 mailing list individuals

^1^Project funded by the Ministry of Science, Research and Art Baden-Wuerttemberg (MWK) “Prevention of sequelae and chronification in Long COVID by developing a regional network with a stepped care concept and piloting a general practice based case management with app (PrELongCOV).” Network development and the SWOT analysis are work packages of the project. The realization was done in cooperation with the Competence Network Preventive Medicine. ^2^Stengel et al. (28).

The network activities are presented in Table 2, which shows many similarities but also differences: In particular, in RN the focus is on the involvement of the specialized sector of the university hospitals, on continuing education and on the supraregional approach, whereas in LU the regional, ambulatory, concrete level of care is visible through the interface agreements and the derivable patients treated in the network. Topics from the stakeholders’ perspective about the patients treated in the network were derived from the advisory board protocols. They describe in terms of severity a wide range from mildly affected with temporary reduction in performance to severely affected with long term suffering. Topics within the described group of severely affected include post-exertional malaise, use of non-established therapies, such as apheresis, and resulting social difficulties up to unemployment due to sickness. Furthermore, a sense of desperation and lack of care was perceived by some stakeholders. The stakeholders reported a high time demands for care. A repeatedly discussed topic were aspects of the psyche in the disease pattern Long-COVID, including the themes patients with and without previous mental illness, overlap with psychosomatic illnesses, and patient concerns about psychologizing. Challenges in attributing the patient-presented symptoms were also repeatedly addressed.

**Table 2:** Network activities.

Competence network Long COVID Rhein-Neckar	Long COVID network Ludwigsburg
**Activities carried out in the network**	
*Web site^1^*	*Web site^2^*
- List of network participants (occupational therapy, speech therapy, neuropsychology, pediatrics, physiotherapy, rehabilitation, self-help, special contact points in inpatient/university settings) - Aims - Information for patients and professionals - Patient pathways - Continuing education - Studies from the network	- List of network participants (general practice with long-COVID specialization, occupational therapy, ENT, cardiology, speech therapy, pediatrics, physiotherapy, pneumology, psychotherapy, rehabilitation, self-help, sports therapy) - Information for citizens and professionals with aims - Patient pathways - Continuing education
*Patient pathways*	*Patient pathways*
- Consensus of a regional care concept based on general practice with 18 experts (multidisciplinary, multispecialty, intersectoral) - Beginning Fall 2021, based on the German S1 Long COVID guideline^3^ (first published Fall 2021)	- Consensus on treatment pathways based on general practice and network interface agreements (multidisciplinary, multispecialty) - Based on the German S1-Long COVID guideline^3^
*Means of communication*	*Means of communication*
- E-mail contact (requests to the network) - E-mail distribution list (availability of network participants) - Newsletter distribution list (external access) - On request, partial dissemination via e-mail distribution lists of institutions (professional associations, Association of Statutory Health Insurance Physicians) - Video conferencing	- E-mail contact option (requests to the network) - E-mail distribution list (availability of network participants) - Video conferencing - Medical care: Telephone, fax
*Regional collaboration*	*Reginal collaboration*
- Initial working groups occupational therapy, physiotherapy (discontinued) - 4 advisory board meetings (online) - Participation of regional stakeholders in training programs - Renewed attempt to set up working groups	- Quality circle (see below) - Within the care by interface agreements - Participation of regional actors in training programs
*Supra-regional collaboration*	*Supra-regional collaboration*
- Repeated informal exchange of experiences with Long-COVID network Ludwigsburg since November 2021 - Exchanges with meso and macro-level with development of follow-up projects, participation in the organization of education, distribution of information and beginning multiplication of regional network structures	- Repeated informal exchange of experience with competence network Long-COVID Rhein-Neckar since November 2021
*Continuing education*	*Continuing education*
- 3 online training, target group: regional physicians - 2 regional trainings for physiotherapists regionally in cooperation with Physio Deutschland (professional association for physiotherapy) - Online-on-demand training, targeted at all health care professionals supra-regional^4^	- 2 regional online trainings - 1 regional online quality circle
**Patients treated in the network**	
*Number*	*Number*
- Cannot be determined from available data	- Total network January–October 2022 approx. 250 patients
*Topics from the stakeholders’ perspective*	
- wide range from mildly affected with temporary reduction in performance to severely affected with long term suffering - severely affected with relevance of topics: - post-exertional malaise - non-established therapies - resulting social difficulties - perceived desperation and lack of care - high time demands for care - challenges in assigning patient-presented symptoms - patients with and without previous mental illness, overlap ping symptoms with psychosomatic illnesses, patient concerns about psychologizing	- 4 general practices January–March 2023 approx. 40 patients, of which 15 are consulted patients from other general practices.

^1^(29), ^2^(30), ^3^(15), ^4^https://drks.de/search/de/trial/DRKS00028869 (last accessed June 8, 2023).

### 3.2. SWOT-analysis

A total of *n* = 14 participants (*n* = 7 RN, *n* = 7 LU) took part in two regionally separated online group discussions. There were *n* = 6 male participants. A more urban place of work was indicated by *n* = 8 and a more rural place of work by *n* = 6. Participants included seven physicians (one outpatient rehabilitation physician, four general practitioners, one pediatrician, one rheumatologist), three occupational therapists, one speech therapist, one physiotherapist, one psychoneurologist and one psychotherapist.

The strengths and weaknesses identified by the participants are presented in Table 3. In both networks, the multidisciplinary-multispecialty character is seen as a strength, in RN also the intersectoral approach. Both networks valued the resulting collaboration. In terms of activities and effects on medical care, differences between the network functions became clear, with RN emphasizing the strength in the area of knowledge transfer and LU emphasizing the strength in the area of concrete contact persons, increasing of caregivers’ motivation, improved interface exchange and more quickly appointments. Regarding the weaknesses mentioned, there was a high level of agreement and consensus about the lack of reimbursement for participation and the limited participation. There was also agreement on the high efforts of care and inadequate reimbursement, as well as the perception that there were still gaps in knowledge and motivation among colleagues. Differences arose in the assessment of collaboration, with RNs reporting a lack of concrete action and realization, which was not an issue in LU.

**Table 3:** Network SWOT analysis—strengths and weaknesses.

Competence network Long COVID Rhein-Neckar	Long COVID network Ludwigsburg
**Strengths (internal) What strengths do we have as a network?**	
*Characteristics* - multidisciplinary-multispecialty networking with personal contacts - intersectoral approach - Network competence *Collaboration* - regional, familiar exchange - concrete interactions - Limited number of stakeholders in the advisory board allows for rapid exchange *Activities* - Fast and regular knowledge transfer - Continuing education structure *Effects on medical care* - Control of patient flows through more precise allocations, achievable to a limited extent/sub-area (e.g., children)	*Characteristics* - multidisciplinary-multispecialty cooperation with therapeutic and medical members *Collaboration* - Working at eye level - Improved exchange between physicians and therapists *Activities* - defined contact persons *Effects on medical care* - Appointments through the network more quickly - Specialist cardiology and pulmonology appointments made easier - Interfaces quickly accessible - Teamwork increases motivation of caregivers *Effects across health systems* - Self-affirmation through improved public perception
**Weaknesses (internal) What weaknesses do we have as a network?**	
*Collaboration* - Still too little exchange and networking - Competence, but lack of concrete approaches for action and realization - Specific issues remain unresolved (e.g., in the area of children/youth) - Limited number of actors with regard to missing disciplines *Network participation* - Voluntary basis of participation - Lack of remuneration for commitment - Limited time commitment to the network *Effects on medical care* - Insufficient remuneration for medical care - Patients and services do not find each other - Persistent knowledge deficits resulting in underuse/misuse of (primary) health care services	*Collaboration* - No personal meeting yet; physical meeting as a goal *Network participation* - Participation rates in exchanges often low - Not all potential actors are reached/involved - Insufficient compensation for reimbursement—high level of private involvement *Effects on medical care* - Network resources do not match needs - Partly unmotivated referral of patients to the network or insufficient clarification of patients. - Lack of feedback from the therapists in some cases - High recording and bureaucratic effort especially for integrative activities

The opportunities and risks identified by the participants are presented in Table 4, which shows a high degree of agreement in the thematic areas. Both networks continued to see a need for intervention in the area of long COVID, with increase of public awareness and mobilization of external funding opportunities seen as relevant. The groups also agreed on the potential transferability of the network approach to other diseases, e.g., Myalgic Encephalomyelitis/Chronic Fatigue Syndrome (ME/CFS), and in LU the promotion of engagement with a “difficult topic” that is often met with rejection was emphasized. In RN, the role of the university hospital in science and networking was seen as an important opportunity, and in LU it was pointed out that experience in the ambulatory sector could support research. Further development opportunities, such as intersectoral care concepts and institutionalization, were also pointed out. Risks identified by both networks ranged from limited resources, such as high effort for integrative care activities to the risk of project termination without sufficient support and recognition. There was a consensus that the network activity could lead to other diseases receiving too little attention. The establishment of alternative care structures was viewed differently, with RNs seeing this as a potential risk and LUs seeing the potential inhibition of such structures as a potential risk. Different lenses were also used to illuminate the issue of mismanagement of care, with attention drawn in RN to the relevance of long COVID subgroups and in LU to the potential mismanagement of patients with other health problems by such a network offering.

**Table 4:** Network SWOT analysis—opportunities and threats.

Competence network Long COVID Rhein-Neckar	Long COVID network Ludwigsburg
Opportunities (external) What opportunities does the environment offer?	
*Long COVID* - Demand still exists - Not a competing player *Effects across health systems* - Public and press relations with resulting in increased awareness of Long COVID and ME/CFS.^1^ - Mobilization of external funding opportunities *Transfer to other diseases* - Transfer to ME/CFS^1^ *Roles/Development* - University Hospital in the role of “Science and Networking” - Joint presentation as “Regional Group - Further development of intersectoral care concepts - Location advantage through the establishment of (intersectoral) networks	*Long COVID* - Structured care - Improved resource allocation - contacts can be reached by externs *Effects across health systems* - Improved external/public awareness and support - Mobilization of external funding opportunities *Transfer to other diseases* - Awareness/support for other fatigue disorders, e.g., ME/CFS^1^ - Encouraging engagement with a “difficult topic” that is often rejected *Roles/Development* - Dynamic adaptability - Networking with other networks - Institutionalization - Experience can support research
Risks (external) What risks does the environment pose?	
*General resources* - Possible creation of competitive structures - Insufficient attention to ME/CFS^1^ and post-COVID-19 vaccination syndrome *Resources of the network* - Limited growth potential as long as resources are limited - Withdrawal of the university sector from coordination/organization *Misuse* - Lack of consideration of Long COVID subgroups	*General resources* - Inhibiting the establishment of alternative care structures - Dependence on the already financially strained health care system - Encouraging the emergence of a counter-movement/rejection - Reduced support for other, competing diseases *Resources of the network* - Increased demand with strain on resources - Unfulfillable, excessive expectations - Limited idealism with risk of project abandonment - Contempt due to preoccupation with Long COVID - Instrumentalization for the use of social services *Misuse* - Mismanagement of patients with other health problems - Risk of fragmentation of care due to specialization

^1^ME/CFS, Myalgic Encephalomyelitis/Chronic Fatigue Syndrome.

## 4. Discussion

The present community case study provided initial insights into the experiences of the Long COVID networks RN and LU, which were established in Southern Germany as *ad hoc* examples of best practice to address the challenges of medical care for Long COVID. In terms of structure and functioning, the networks agreed on a primary care-based, multidisciplinary and multispecialty approach. The main differences in realization emerged in LU as a focus on the ambulatory setting with more concrete and specifically coordinated agreements that could be applied to the individual patient in the medical care between actors. In contrast, RN showed a focus on the intersectoral character with the involvement of the specialized sector of university hospitals, knowledge transfer and the supra-regional approach with the involvement of the meso and macro level. The SWOT analysis from the perspective of the ambulatory network actors followed the structure and functioning of the networks. It showed that first steps of an internationally and nationally recommended multidisciplinary multispecialty approach (18, 19, 24, 25, 37) could be realized through the network intervention, despite the limited resources and short duration, up to motivational effects for dealing with an “unpopular” topic and positive effects on concrete cooperation. This could have the potential to help fill an identified gap in care for an underserved group (8, 9), but limitations such as insufficient resources and other threats were also highlighted. The exemplary application in Long COVID showed potential for transferability to other diseases such as ME/CFS and further development in the area of intersectoral care models.

According to Mitchell, networks are defined “[…] as a specific set of linkages among a defined set of persons, with the additional property that the characteristics of these linkages as a whole may be used to interpret the social behavior of the persons involved” (38). In the present results, the reported network mechanisms, especially in LU, showed hints of “navigation.” There were also hints of “contagion” promoting factors, such as increased motivation, or “contagion” inhibiting factors, such as disdain, and hints of “negotiation,” i.e., the adoption of ideas, attitudes and behaviors (39, 40), e.g., in the use of developed care pathways. This is in line with the findings on coping with the acute COVID pandemic in primary care, where belonging to networks was found to be helpful (41) and contributed to the resilience (42) of the primary care system (43). However, even outside the pandemic, participation in primary care networks has been shown to be a motivating factor for guideline-based care and adoption of new routines (44).

The primary care-based, stepped approach in the networks is in line of guidelines (7, 14, 15). There is an urgent need for education and training in post-infectious diseases (11). Such educational opportunities were offered in both networks and were particularly expanded in the intersectoral network. It is known from implementation science that education is a key component of implementing innovations, but that other strategies for behavior change need to be added (45). For example, including role models (46), communicating the relevance of the issue in the region, and peer-to-peer learning can increase the impact of training (45), and hints of such a realization was found in the results for all of the above.

The absolute number of patients with long COVID treated per general practitioner in Germany is low, but also limited in the specialist ambulatory setting (47) and may explain the knowledge deficits reported by patients but also by health care providers (8, 9, 48). In settings where many patients are seen in a short period of time, such as university-based specialty ambulatory clinics, a faster learning curve can be expected due to the number of patients, analogous to learning procedures where a certain number of examinations are required to achieve diagnostic confidence (49). The iterative processes and interconnectedness at and between different levels in the networks could contribute to building a learning health system that can respond and disseminate knowledge quickly and adaptively (20). In this context, the aspect mentioned in the SWOT analysis that experience in the ambulatory network can also contribute to research suggests interactions in knowledge transfer between sectors, especially since primary care physicians typically know their patients, their history and their course (50). Furthermore, the model of practice-based evidence could also be applied in the network as a complement to evidence-based practice (51).

The transferability of the intervention experience to other post-infectious diseases, as indicated by the results, seems obvious, especially since there is a subgroup with criteria of ME/CFS described after many infectious diseases (10, 52), as well as their underuse (53). Again, multidisciplinary-multispecialty approaches are recommended by international bodies (54). The optimal integration of the ambulatory care system, which has been developed nationwide in Germany, into a stepped concept could provide high and dynamic care capacities, leaving room for university ambulatory clinics to fulfill the tasks of “teaching, research and care of complex cases” according to § 117 SGB V.

### 4.1. Strengths and weaknesses

A strength of the study is the written presentation of identified themes and strategic need for action of the realized networks as *ad hoc* best practice examples despite limited resources in terms of time and content. The pragmatic and quick approach of document analysis and SWOT analysis with an explorative character offers the possibility to quickly generate initial strategic hypotheses on the topic of long COVID networks.

Document analysis has advantages such as being an efficient method, availability and cost effectiveness, and limitations such as lack of detail, low retrievability and biased selectivity (32). The potential bias of using the people involved in the coordination was countered by the exchange with LG as an independent member of the study team.

The SWOT analysis is a method for strategy development, but it does not replace established social network analysis or quantitative or qualitative methods at the patient, professional (inpatient and outpatient) and system levels, which should follow in the next step. Due to the study design, the present work does not claim to be representative. The number of participants (*n* = 14) is limited, but at the same time the statements included a broad spectrum, as urban and rural workplaces, men and women, different professional groups and two different regions in southern Germany were surveyed. A selection bias may have occurred due to the participation of particularly motivated or positively minded individuals. The invited patient representatives from the advisory board of the RN network could not participate due to time or health reasons. One-sided data collection from stakeholders can lead to bias, so the results must be clearly interpreted as a survey from their perspective. Including patients’ experiences is important (8), and should be considered in future studies.

### 4.2. Implications

A SWOT analysis is used to identify strategic needs for action (30). The results of this process in the described regional networks showed on the one hand indications that network participation could contribute to a rapid learning and resilient health system coping with long COVID, for example through the reported resulting collaboration, contribution to knowledge transfer, and improvement of care for individual patients. On the other hand, at the same time, barriers such as lack of reimbursement, high efforts of care, and persistent motivation gaps became apparent. Potentials mentioned were, e.g., transferability to other diseases such as ME/CFS, promotion of addressing a “difficult topic” and promotion of intersectoral care concepts; risks mentioned were, e.g., limited network resources and negative effects on the development of other structures. Resulting implications for practice and research address a call to policy makers and funders to support further research to find out what generalizable results regarding usefulness, effectiveness, and efficiency including transferability to other post-infectious diseases can be derived, what aspects best contribute to impact, what is needed for the sustainable establishment, and, in summary, generate more robust evidence. Because they are different, the pros and cons of both networks need to be considered. The application of participatory approaches involving patients and stakeholders seems reasonable and timely (55).

## 5. Conclusion

Given the scientific reports of post COVID as a long-lasting condition with heterogeneous symptoms, early detection and prevention are important for healthcare systems (56). As an *ad hoc* best practice example to contribute to an area-wide and continuous care, two multidisciplinary and multispecialty Long COVID networks – one intersectoral also—were established, integrating the ambulatory sector. A SWOT analysis emerged hints of potential to improve care for Long COVID and other conditions such as ME/CFS and other post-infectious diseases. At the same time, pitfalls and possible solutions were identified. Overall, there is potential for further development of Long COVID networks including the derivation of generic findings on intersectoral care models and health system resilience, which should be accompanied by health services research and requires financial support to be feasible.

## Data availability statement

The complete data cannot be made publicly accessible due to the assured data protection regulations. Reasonable requests to access these datasets should be directed to the corresponding author.

## Ethics statement

The studies involving humans were approved by the Ethics Committee of the Medical Faculty Heidelberg. The studies were conducted in accordance with the local legislation and institutional requirements. The participants provided their written informed consent to participate in this study.

## Author contributions

SS, JS, and UM conceptualized the long COVID network Rhein-Neckar. SS, LG, SV, JKoe, and UM conceptualized and designed the study. SS, LG, JKol, SV, and KT performed the data collection. SS and LG performed the data analysis. SS prepared the tables and drafted the first version of the manuscript. SS, LG, JKol, KT, SV, JKoe, JS, and UM performed data interpretation. All authors participated in a critical revision of the manuscript and approved the final version of the manuscript.
